# The Anthropocene – The biggest threat to health on the African continent

**DOI:** 10.4102/phcfm.v11i1.2151

**Published:** 2019-09-16

**Authors:** Robert Mash

**Affiliations:** 1 Division of Family Medicine and Primary Care, Stellenbosch University, Cape Town, South Africa

In the last few months, we have seen an escalation in environmental disasters, stringent warnings and political awakenings to the scale of the imminent environmental crisis facing us and our only home – our planet. On the East coast of Africa, we have seen Beira in Mozambique devastated by Cyclone Idai, maybe the first city in the world to be devastated by climate change, deforestation and sea-level rise.^1^ This was closely followed by Cyclone Kenneth in Tanzania and Mozambique as well as flooding in Durban, South Africa, that also caused many deaths. On the west coast of Africa, Namibia has just declared a national emergency because of drought and Cape Town is still recovering from its own water crisis.

The environmental crisis is created by the impact of rapidly growing human populations and activities on the planetary ecosystems. The fact that humans are impacting the planet on a geological scale has led scientists to label the period we now live in as the anthropocene.^2^ We have literally become a force of nature.

The environmental crisis facing communities across the planet is, in fact, a series of crises that are coming together in a perfect storm. Climate change has had the most visible coverage in the media, but we are also seeing biodiversity loss on an unprecedented scale. One million species across the planet face extinction at a rate hundreds of times higher than in the last 10 million years.^3^ We depend on these ecosystems for our own existence in terms of, for example, food and clean water. Deforestation is a major contributor to loss of biodiversity and climate change. Meat consumption and use of land to support livestock and farming of monocultures underlies deforestation and in the oceans, fish stocks are depleted. Accompanying this is pollution on a massive scale. Many cities and regions struggle with air pollution, while plastic enters our water and food supply and pollutes our oceans.

Young people have seen the crisis, which threatens their future well-being.^4^ In London, the recent extinction rebellion disrupted business as usual and helped to motivate Parliament to declare climate change as a national emergency.^5^ Young people have led protest actions across the world. Many religions have spoken out including the Pope^6^ and most recently, the Anglican church has recognised climate change as a global emergency.^7^ We have only a decade to transform our relationship to the planet if we want to avoid extreme climate change.^8^

In Africa, the environmental crises have had much less traction amongst politicians and civil society. In the recent South African elections, the issue is barely mentioned or understood in the political party manifestos and despite South Africa’s large ecological footprint (carbon emissions are more than the United Kingdom [UK]), we are not taking the issues seriously enough.^9^

Many African countries are still struggling to build a strong social foundation for their populations (e.g. health, employment, housing, education and security) and have contributed little to problems such as climate change. Worrying about the transgression of environmental limits is not on the political agenda. It is likely, however, that less resilient communities in low- and middle-income African countries will suffer the most from the environmental crises. As African countries develop, we will need to follow a different trajectory that takes these environmental limits into account. It is not possible to negotiate or compromise politically with the dynamics of physics and ecology.

The consequences for people’s health and health systems are also enormous. We can expect an escalation in environmental disasters such as floods, droughts and fires that will stretch our emergency medical services and disaster relief agencies. Such disasters are accompanied by injuries, psychological trauma, outbreaks of infectious diseases such as cholera, disruption of health services (such as for HIV and obstetrics) and displacement of people. Food insecurity will be increased with implications for malnutrition and child health. Safe potable water supplies may be disrupted. Air pollution impacts on cardiovascular and respiratory health as well as allergies. Certain vectors and infectious diseases will shift or increase their coverage as habitats and climate change.

Strong primary healthcare systems and emergency medical services can help communities to withstand the impact of the environmental crises.

As family medicine and primary care providers, we need to speak out about the environmental crises facing the populations that we serve and add our voice in order to create urgency amongst politicians and policy makers. We do not have much time to transform our relationship with the planet and change our patterns of consumption. Health professionals can help lead. Consider joining physicians for planetary health.^10^

We need to fundamentally change our individual and collective behaviours. This also applies to health services and systems that contribute to the environmental crises. The Global Green and Healthy Hospitals’ network^11^ has defined 10 areas in which health services can respond: leadership, energy use, water use, waste management, transport, pharmaceuticals, chemicals, procurement, buildings and food. Consider joining the network.

As individuals, we also need to change in similar ways:^12^

- Eating a healthy and balanced diet with a larger proportion and a wide variety of plant-based foods, only buying meat and fish from sustainable sources, and not wasting food, all help to protect our planet.
- Re-use, buy less, and choose nature-friendly products. For example, only buy paper and wood from recycled or sustainable sources featuring the Forest Stewardship Council (FSC) logo to be confident you’re not harming the world’s forests.
- Help combat climate change, and increase air quality by reducing your use of fossil fuels and supporting renewable energy; for example, in the way you get around and power your home.
- Our voices are powerful. Use yours to call for nature-friendly choices. For example, ask your supermarket to stop using plastic bags, avoid plastic packaging and ask food companies to only use palm oil that is certified sustainable, and hasn’t caused deforestation.
- Support local wildlife and encourage nature to grow, from beach clean ups to urban gardens, support local initiatives that protect and restore nature.
